# Ameliorative effect of flavocoxid on cyclophosphamide-induced cardio and neurotoxicity via targeting the GM-CSF/NF-κB signaling pathway

**DOI:** 10.1007/s11356-022-20441-5

**Published:** 2022-05-16

**Authors:** Fatma F. Elsayed, Waad M. Elshenawy, Eman M. Khalifa, Mohamed R. Rizq, Rania R. Abdelaziz

**Affiliations:** Department of Pharmacology & Toxicology, Faculty of Pharmacy, Mansoura University, Mansoura, 35516 Egypt

**Keywords:** Neurotoxicity, Cyclophosphamide, Flavocoxid, GM-CS, Cardiac injury, NF-κB

## Abstract

Cyclophosphamide (Cyclo) is a chemotherapeutic agent used as an immunosuppressant and as a treatment for many cancerous diseases. Many previous pieces of literature proved the marked cardio and neurotoxicity of the drug. Thus, this research provides evidence on the alleviative effect of flavocoxid on the cardiac and brain toxicity of cyclophosphamide in mice and determines its underlying mechanisms. Flavocoxid (Flavo) is a potent antioxidant and anti-inflammatory agent that inhibits the peroxidase activity of cyclooxygenase (COX-1 and COX-2) enzymes and 5-lipooxygenase (*5-LOX*). Flavo was administered orally (20 mg/kg) for 2 weeks, followed by Cyclo (100 mg/kg, i.p.) on day 14. Higher heart and brain weight indices, serum lactate dehydrogenase (LDH), creatine kinase (CK-MB), and nitric oxide (NO) were mitigated following Flavo administration. Flavo modulated oxidative stress biomarkers (malonaldehyde (MDA), glutathione (GSH), and superoxide dismutase (SOD)), tumor necrosis factor-α (TNF*-*α), and interleukin (IL)-1β. Additionally, cardiac troponin I (cTn-I), nuclear factor kappa B (NF-κB), brain amyloid precursor protein (APP), and granulocyte macrophage colony-stimulating factor (GM-CSF) were decreased by Flavo administration. Moreover, Flavo ameliorated heart and brain histopathological changes and caspase-3 levels. Collectively, Flavo (20 mg/kg) for 14 days showed significant cardio and neuroprotective effects due to its antioxidant, anti-inflammatory, and antiapoptotic activities via modulation of oxidative stress, inflammation, and the GM-CSF/NF-κB signaling pathway.

## Introduction

Cyclophosphamide (Cyclo) is a potent alkylating cytotoxic agent used as a treatment for various types of cancer. Additionally, it is a potent immunosuppressant used for organ transplantation and autoimmune diseases (Chen et al. 2017; Elmariah et al. 2018). Cyclo exerts its antineoplastic cytotoxic effects through its active metabolite, phosphoramide. At the same time, acrolein is the metabolite that is mainly responsible for Cyclo-induced toxicities, including cardiotoxicity, nephrotoxicity, hepatotoxicity, male reproductive toxicity, teratogenicity, and neurotoxicity (Avci et al. 2017; Zhai et al. 2018). Clinically, Cyclo-induced cardiotoxicity is dose-dependent and is characterized by acute onset (Goldberg et al. 1986; Braverman et al. 1991). Cyclo has also been reported to cause brain toxicity in animal studies, but clinically, it has not been well estimated (Chen et al. 2019). Furthermore, several studies have showed a massive elevation of reactive oxygen species (ROS) after Cyclo administration. This increase in ROS damages cellular biomolecules, leading to cell death (Aladaileh et al. 2019). Additionally, Cyclo can induce toxicity through the depletion of cellular decreased glutathione (GSH) and superoxide dismutase (SOD), thus leading to the upregulation of MDA and increased nitric oxide (NO), resulting in multiorgan hazardous side effects due to oxidative stress (Moghe et al. 2015; Aladaileh et al. 2019). Moreover, Cyclo can form a protein adduct in cardiomyocytes through its toxic metabolite acrolein, leading to NF-κB activation, the main inducer of proinflammatory gene induction, and functions. After translocation to the nucleus, activated NF-κB transcript the production of inflammatory cytokines, such as interleukin 1β (IL-1β) and tumor necrosis factor-α (*TNF-α*), elevating their levels (Iqubal et al. 2019a). However, some studies showed that Cyclo could cause marked neurotoxicity (Rzeski et al. 2004; Seo et al. 2019). Cyclo can cause a significant oxidative imbalance in the brain, which can be lowered by the coadministration of free radical scavengers (Singh and Kumar 2019). Thus, in the current research, Flavo is used as an antioxidant agent with Cyclo to reduce its toxicity.

Flavocoxid (Flavo) is a medical food consisting of purified plant-based bioflavonoids, including baicalin, extracted from *Scutellaria baicalensis*, and catechin, from *Acacia catechu*, with a concentration higher than 90% purity (Burnett et al. 2011). Flavo causes balanced inhibition of both cyclooxygenase-1 (*COX-1*) and *COX-2* peroxidase enzyme activity and shows significant potent inhibition of 5-lipooxygenase (*5-LOX*) (Altavilla et al. 2009; Burnett et al. 2011). Additionally, it has anti-inflammatory and immunomodulatory activities and is a potent free radical scavenger (Messina et al. 2009; Polito et al. 2010; Bitto et al. 2012). Flavo downregulates inflammatory markers not only at the protein level but also at the gene expression level. Moreover, Flavo can lower oxidative imbalance by inhibiting NF-κB, the primary mediator of proinflammatory gene induction, and functions. As a result, Flavo can downregulate the gene expression of *COX-2*, *5-LOX*, *iNOS*, and *TNF-α*, limiting their levels and their metabolites, including prostaglandin E2 (PG-2), leukotriene B4 (LTB4), and NO (Altavilla et al. 2009; Burnett et al. 2011).

The current study was conducted to assess the potential beneficial effect of flavocoxid, a selective *COX-1*/*COX-2* peroxidase inhibitor, on the progression of cardio and neuroinjury and its associated pathological and biochemical consequences in an experimental model of cyclophosphamide-induced cardio and neurotoxicity in mice.

## Materials and methods

### Chemicals and treatments

Cyclo was bought from Astra medica co. Egypt as “Endoxan (1 g)” and was diluted with distilled water and injected intraperitoneally. Flavo was bought from MEDVANTX, USA, as “Limbrel 250™” and was suspended in carboxymethyl cellulose (CMC, El-Nasr co. Egypt) and taken orally. Thiopental sodium, acetic acid, AA (20%), KCL, and thiobarbituric acid, Na dodecylsulfate, pyrogallol, tris buffer, phosphate buffer, TCA-EDTA, and Elman’s reagent were obtained from Sigma Aldrich Chemical Co. (St. Louis, MO, USA).

### Animals

Forty male Swiss albino mice weighing approximately 20–25 g were purchased from the “Egyptian Organization for Biological Products and Vaccines,” Giza, Egypt. They were kept in standard animal care efficiency (animal house, Faculty of Pharmacy, Mansoura University, Egypt). Experimental procedures were approved by the “Research Ethics Committee,” Faculty of Pharmacy, University of Mansoura, Egypt.

### Study design

One week passed on acclimation of the mice, and then, they were divided into four groups (*n* = 10 mice).

1. The control group was provided with the solvent (0.5% CMC) from day 0 to day 14.
2. Administration of Flavo (20 mg/kg) orally for 2 weeks.
3. The Cyclo group was injected with Cyclo (100 mg/kg, i.p.) as a single dose on day 14.
4. The protected group was given Flavo from day 0 to day 14 with Cyclo (100 mg/kg) as a single dose on day 14 (Abdelaziz et al. 2018).

### Blood sampling

Before sacrificing, blood samples were collected from the retro-orbital sinus, and sera were separated and stored at − 80 °C to estimate LDH, CK-MB, and NO.

### Heart and brain weight/body weight ratio determination and heart and brain homogenate preparation

Mice were sacrificed under mild thiopental anesthesia (50 mg/kg, i.p.), and then, whole hearts and brains were removed, cleaned with ice-cold 1.15% potassium chloride (KCl) at pH 7.45, and scaled. Then, the heart and brain weight per body weight ratio was measured. Chilled KCl was applied to the first portion of the heart and brain tissue for homogenization using a glass homogenizer on ice to prepare 10% w/v tissue homogenates. After that, the homogenate was centrifuged at 4000 rpm and 4 °C; the supernatant was detached and finally isolated into aliquots and frozen at − 80 °C (Maresh et al. 2020).

### Estimation of serum biochemical parameters

#### Lactate dehydrogenase

LDH was determined quantitatively following the method of Henry (1974). LDH is responsible for the conversion of pyruvate to L-lactate oxidizing NADH, which absorbs light at 340 nm. NADH oxidation is directly proportional to the catalytic activity of LDH.

#### Creatinine kinase

CK-MB was quantitatively assessed following the method of Wurzburg et al. (1977). The antibody inhibits subunit M of CK-MB. The noninhibited CK-MB undergoes a series of reactions, producing NADPH, which is measured photometrically. CK-MB was calculated as unit per liter (Würzburg et al. 1977).

#### Nitric oxide

In an acidic medium and in the presence of nitrite, one of the final two nitric oxide products, the formed nitrous acid diazotizes sulfanilamide, and the product was complex with V- (naphthyl) ethylene diamine. The resulting bright reddish-purple azo dye can be measured at 540 nm (Amin et al. 2020).

### Estimation of brain and heart oxidative stress

#### Heart and brain malonaldehyde level

MDA content was estimated using the method of Ohkawa et al. (1979). Thiobarbituric acid reactive substances were estimated as MDA. MDA was expressed as nanomole/gram wet tissue at 532 nm.

#### Heart and brain glutathione level

GSH level was assessed using the method of Ellman (1959). GSH was expressed as micromole/gram wet tissue at 412 nm.

#### Heart and brain superoxide dismutase level

SOD activity was determined using the method of Marklund and Marklund (1974). SOD activity was expressed as U/gram tissue at 420 nm.

### Estimation of heart and brain tumor necrosis factor-α and interleukin-1β

The TNF-α and IL-1β kits are sandwich enzyme immunoassay for in vitro quantitative measurement of *TNF-α* and IL-1β (eBioscience, USA).

### Estimation of cardiac troponin I and brain granulocyte macrophage-colony stimulating factor in heart and brain homogenates

cTn-I and GM-CSF levels were determined using a double-antibody sandwich ELISA kit procured from Biocodon Technologies, USA.

### Western blot analysis

The expression levels of nuclear factor kappa B (NF-κB) and amyloid precursor protein (APP) in heart and brain tissues, respectively, were evaluated using western blot analysis. A Bradford Protein Assay Kit (SK3041; Markham Ontario L3R 8T4 Canada) was used to run the protein concentration assay. A total of 20 µg of protein in each sample was loaded and separated using electrophoresis on a polyacrylamide gel (TGX Stain-Free™ FastCast™ Acrylamide Kit (SDS-PAGE) and then transferred onto a PVDF membrane. The membrane was blocked with Tween 20 (TBST) buffer and 3% bovine serum albumin (BSA) at 25 °C for 1 h. Primary antibodies of APP and NF-κB were bought. Incubation was performed overnight in each primary antibody solution against the blotted target protein at 4 °C (Cell Signaling Technology). The blot was then washed and incubated with HRP-conjugated secondary antibody (goat antirabbit IgG-HRP-1 mg Goat mab-Novus Biologicals). A chemiluminescence substrate was detected by antibody interaction (ClarityTM Western ECL substrate Bio-Rad cat#170–5060). A CCD camera–based imager was used to capture the chemiluminescent signals. Software of image analysis was used to read the band intensity of the target proteins against control sample beta actin (housekeeping protein) by protein normalization on the ChemiDoc MP imager.

### Histopathological examination

A small second portion of heart and brain tissues of mice were histopathologically figured out. Samples were settled with 10% formalin buffer for 24 h at 25 °C before they were fixed in paraffin and sliced (3–5 µm). Finally, H&E staining was performed, and the slides were seen in a blind manner (Maresh et al. 2020).

### Immunohistochemical examination of cardio and neuroapoptosis (caspase-3)

Immunohistochemical examination was applied for a sensitive marker for apoptosis (caspase-3) following the manufacturer’s instructions (Biocare Medical, Pacheco, CA, USA). The paraffin was segregated from the paraffin-embedded third portion of the heart and brain sections before the endogenous peroxidase activity was frustrated with 3% H_2_O_2_. Citrate buffer used in antigen retrieval achievement. Then, 5% BSA was conducted on the heart and brain sections to remove any nonspecifically bound protein after cooling. Then, the sections were brooded overnight (4 °C) with primary antibody (rabbit monoclonal anticaspase-3). PBS cleaned the sections, afterward; they were incubated with a secondary conjugated polymer for 30 min at RT on the next day. The next step was incubation for 5 min RT with Biocare’s 3,3-diaminobenzidine (DAB) or for 6–8 min at 25 °C with Biocare’s Warp Red Counterstain with Mayer’s hematoxylin, followed by soaking with deionized water. Tacha’s Bluing Solution was performed for 1 min, followed by wetting with deionized water. Biocare’s MACH 4 detection system was employed for standardizing this antibody. Pictures of sections were taken under a microscope (Nikon) (Gown and Willingham 2002).

### Statistical analysis

Data (means ± SEM) are shown. Statistical graphing and analysis were conducted using GraphPad Prism 6 software (CA, USA). The significance level was set at *p* < 0.05. The statistical difference in the results was determined using one-way analysis of variance (ANOVA), followed by Tukey–Kramer test. The study of immunohistochemistry scores was conducted using a nonparametric Kruskal–Wallis test, followed by Dunn’s multiple comparison post hoc test.

## Results

### Influence of Flavo on heart and brain indices in Cyclo-injected mice

There was no significant difference in heart weight index between all groups, and in the brain, Cyclo increased its weight index by 22.22% and 33.33% compared to the control and Flavo groups, respectively. Flavo in treated group reduced the brain weight index by 11.11% compared to the Cyclo group (Table 1).

**Table 1:** Effect of Flavo on heart and brain weight indices in cyclo-injected mice

Group	Heart weight index	Brain weight index
Control	0.007 ± 0.002	0.014 ± 0.0005
Flavo	0.005 ± 0.0014	0.012 ± 0.002
Cyclo	0.005 ± 0.0004	0.018 ± 0.0008*$
Flavo + Cyclo	0.0057 ± 0.0009	0.016 ± 0.0018#$

Administration of (20 mg/kg) P.O Flavocoxid (flavo) to mice from day 0 to 14 followed by Cyclophosphamide (Cyclo) i.p (100 mg/kg) at day 14. Data were expressed as mean ± SED (*n* = 10). *, $, #, significantly different when compared to normal control, Flavo, and Cyclo groups, respectively, using one-way ANOVA test with Tukey Kramer post hoc test (*p* < 0.05)

### Influence of Flavo on serum biochemical parameters (LDH, CK-MB, and NO) in Cyclo-injected mice

After the administration of Cyclo (100 mg/kg, i.p.) injection, there were significant increases in LDH, CK-MB, and NO by 228%, 113%, and 85%, respectively, compared with those in the control group. However, serum LDH, CK-MB, and NO in the protected group significantly decreased by 59%, 59%, and 61%, respectively, compared with the Cyclo-treated group. Oral Flavo alone caused no significant difference in the measured biochemical parameters compared with those of the control group (Table 2).

**Table 2:** Effect of Flavo on heart and brain weight indices in cyclo-injected mice

Groups	LDH (U/L)	CK-MB (U/L)	NO (µmol/L)
Control	2886.25 ± 232.87	273.75 ± 14.097	40.75 ± 0.853
Flavo	3444.5 ± 218.5	305 ± 5.00	50.5 ± 1.500
Cyclo	9480.5 ± 89.553*$	584.25 ± 21.301$	75.25 ± 4.837*$
Cyclo + Flavo	1488.19 ± 107.67*$#	240.8 ± 16.3111#	29.4 ± 2.112$#

Oral administration of (20 mg/kg) Flavocoxid (Flavo) to mice from day 0 to 14 followed by Cyclophosphamide (Cyclo) intraperitoneal i.p (100 mg/kg) at day 14. Data were expressed as mean ± SED (*n* = 10). *, $, #, significantly different when compared to normal control, Flavo and Cyclo groups, respectively, using one-way ANOVA test with Tukey Kramer post hoc test (*p* < 0.05)

### Influence of Flavo on heart and brain oxidative stress biomarkers in Cyclo-injected mice

**Fig. 1:**
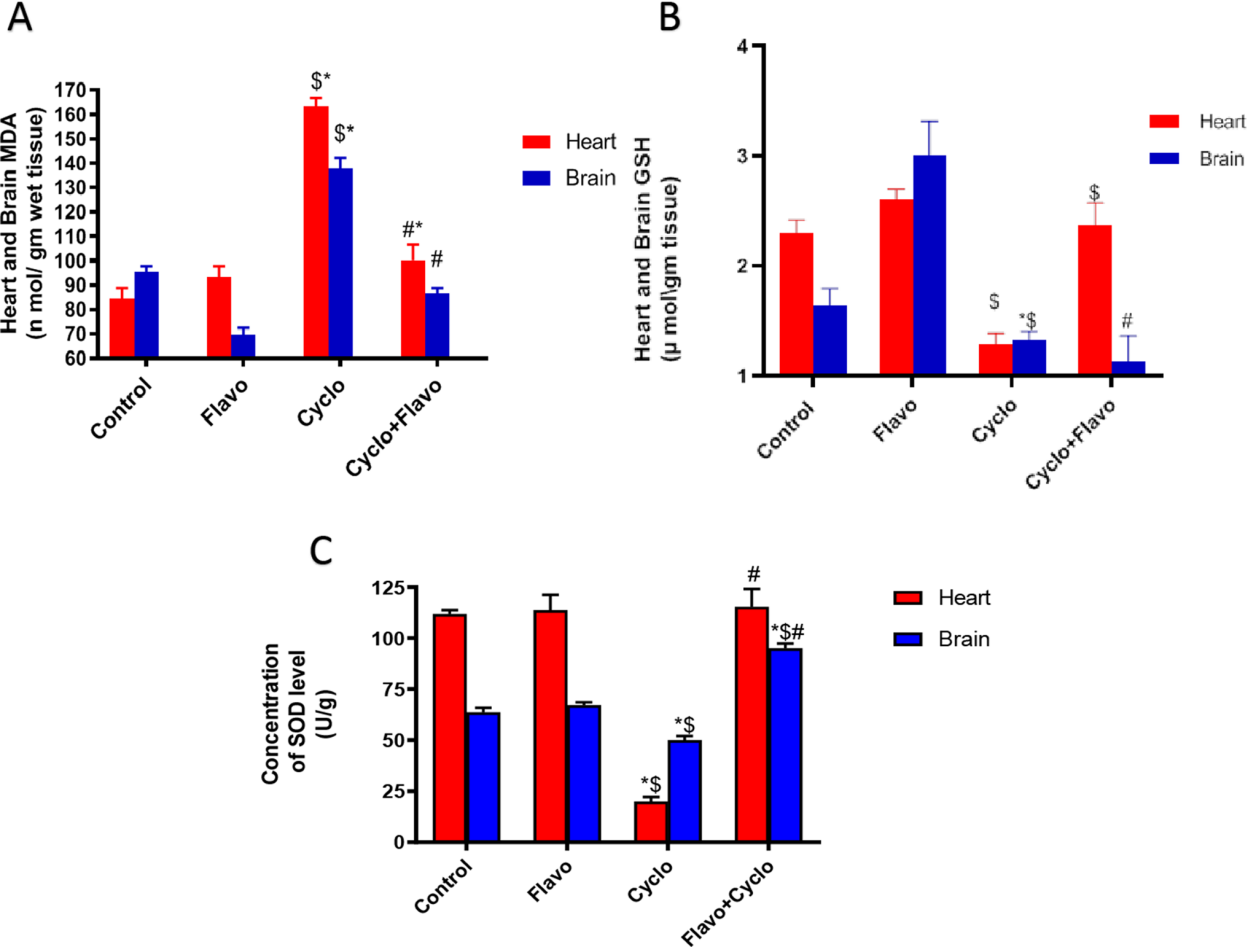
Effect of Flavo on oxidative stress biomarkers in Cyclo-injected mice. Oral administration of (20 mg/kg) flavocoxid (Flavo) to mice from day 0 to 14 followed by cyclophosphamide (Cyclo) intraperitoneal (100 mg/kg) on day 14. Data were expressed as mean ± SED (*n* = 10). ^*, $, #^Significantly different compared to normal control, Flavo, and the Cyclo groups, respectively, using one-way ANOVA test with Tukey–Kramer post hoc test (*p* < 0.05). (**A**) MDA, (**B**) GSH, and (**C**) SOD

- Influence on MDA
  Cyclo significantly increased MDA levels in heart homogenates by 48% and 42.2% compared to the control and Flavo groups, respectively, and in brain homogenates by 30% and 49.7% compared to the control and Flavo groups. On the other side, Flavo in the protected group decreased MDA level by 38% and 37% in the heart and brain, respectively, compared to the Cyclo group (Fig. 1A).
- Influence on GSH
  Cyclo markedly depleted the GSH level in heart homogenates by 79% and 103% compared to the control and Flavo groups, respectively. However, Flavo increased GSH levels in the heart by 85% compared to the Cyclo group, and there is no significant difference in brain GSH compared to the Cyclo group (Fig. 1B).
- Influence on SOD
  Cyclo decreased heart SOD level significantly by 461% and 471% compared to the control and Flavo groups, respectively, and in brain homogenates similarly by 27% and 43%. Flavo increased its level markedly by 479.3% and 90% in heart and brain homogenates, respectively, compared to the diseased group (Fig. 1C).

### Influence of Flavo on heart and brain TNF-α and IL-1β in Cyclo-injected mice

**Fig. 2:**
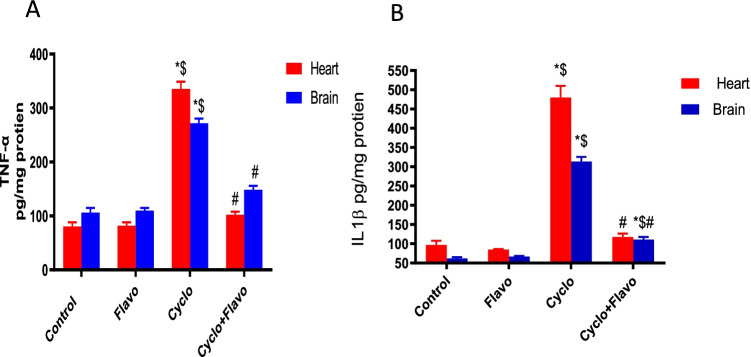
The effect of Flavo on TNF-α and IL-1β in Cyclo-injected mice. Oral administration of (20 mg/kg) flavocoxid (Flavo) to mice from day 0 to 14 followed by cyclophosphamide (Cyclo) intraperitoneal (100 mg/kg) at day 14. Data were expressed as mean ± SED (*n* = 10). ^*, $, #^Significantly different compared to normal control, Flavo, and the Cyclo group respectively, using one-way ANOVA test with Tukey–Kramer post hoc test (*p* < 0.05). (**A**) Tumor necrosis factor-α (TNF-α) and (**B**) interleukin (IL) 1β

Cyclo significantly elevated *TNF-α* in heart and brain homogenates by 4.25- and 2.6-fold, respectively, compared with the healthy control group. However, Flavo in the protected group decreased *TNF-α* level by 69% and 45% in heart and brain homogenates, respectively, compared to the diseased group (Fig. 2A). Regarding IL-1β, Cyclo markedly increased its level in heart homogenates by 79.7% and 82% compared with the control and Flavo groups, respectively, and similarly in the brain by 80% and 78.6%. In the Flavo group, there was a marked decline in IL-1β level by 75% and 64.5% in the heart and brain homogenates, respectively, compared to the diseased group (Fig. 2B).

### Influence of Flavo on cTn-I and brain GM-CSF in Cyclo-injected mice

In the Cyclo group (100 mg/kg), there were significant increases in heart troponin I by 84.3% and 84.35% compared with the control and Flavo groups, respectively. However, Flavo protection (20 mg/kg, orally) decreased this level by 26.2% compared with the Cyclo-injected group (Table 3).

**Table 3:** Effect of Flavo on cardiac troponin I (cTn-I) and brain GM-CSF in Cyclo injected mice

Groups	Heart troponin I (pg/mg protein)	Brain GM-CSF (pg/mg protein)
Control	48.5 ± 4.2	19.4 ± 1.9
Flavo	48.3 ± 0.2	18.35 ± 0.35
Cyclo	260.46 ± 9.1*$	54.73 ± 0.35*$
Cyclo + Flavo	92.5 ± 10.752#	25.9 ± 3.1#

Oral administration of (20 mg/kg) Flavocoxid (flavo) to mice from day 0 to 14 followed by Cyclophosphamide (Cyclo) intraperitoneal i.p (100 mg/kg) at day 14. Data were expressed as mean ± SED (*n* = 10). *, $, #, significantly different when compared to normal control, Flavo, and Cyclo groups, respectively, using one-way ANOVA test with Tukey Kramer post hoc test (*p* < 0.05)

After the administration of Cyclo (100 mg/kg), there were significant increases in GM-CSF by 73.82 and 74.89 compared with the control and Flavo groups, respectively. Administration of Flavo (20 mg/kg, orally) before Cyclo significantly decreased GM-CSF by 32.12 compared with the Cyclo-injected group. Oral Flavo alone caused no significant difference in measured biochemical parameters compared with those of the control group (Table 3).

### Influence of Flavo on heart NF-κB and brain APP in Cyclo-injected mice

**Fig. 3:**
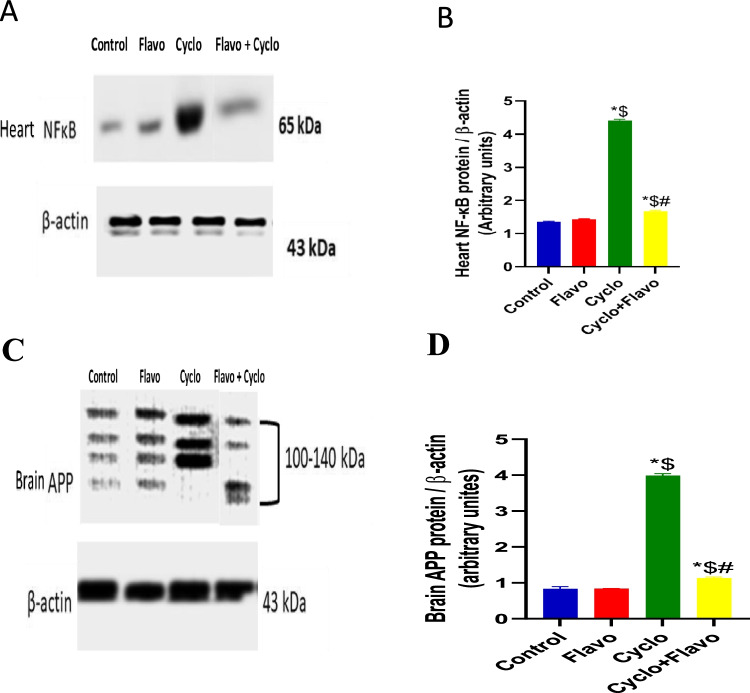
The effect of Flavo on heart NF-κB and brain APP in Cyclo-injected mice. Oral administration of (20 mg/kg) flavocoxid (Flavo) to mice from day 0 to 14 followed by cyclophosphamide (Cyclo) intraperitoneal (100 mg/kg) on day 14. Data were expressed as mean ± SED (*n* = 10). ^*, $, #^Significantly different when compared to normal control, Flavo, and Cyclo group respectively, using one-way ANOVA test with Tukey–Kramer post hoc test (*p* < 0.05). Western blots bands of (**A**) Heart NF-κB and (**C**) Brain APP. Densitometric analyses of relative band intensities. (**B**) Nuclear factor kappa B (NF-κB) and (**D**) amyloid precursor protein (APP)

Compared to that in the control group, Cyclo caused a marked elevation in the activity of NF-κB, and the administration of Flavo caused significant suppression of the activity of this complex protein by 61% compared to the Cyclo group (Fig. 3A and B).

A marked increase in APP level was observed in the Cyclo-administrated group compared with that in the control group. By contrast, there was a clear decrease in brain APP level when administering Flavo compared to Cyclo by 71% (Fig. 3C and D).

### Influence of Flavo on cardiac and brain histopathological changes in the Cyclo-injected group

**Fig. 4:**
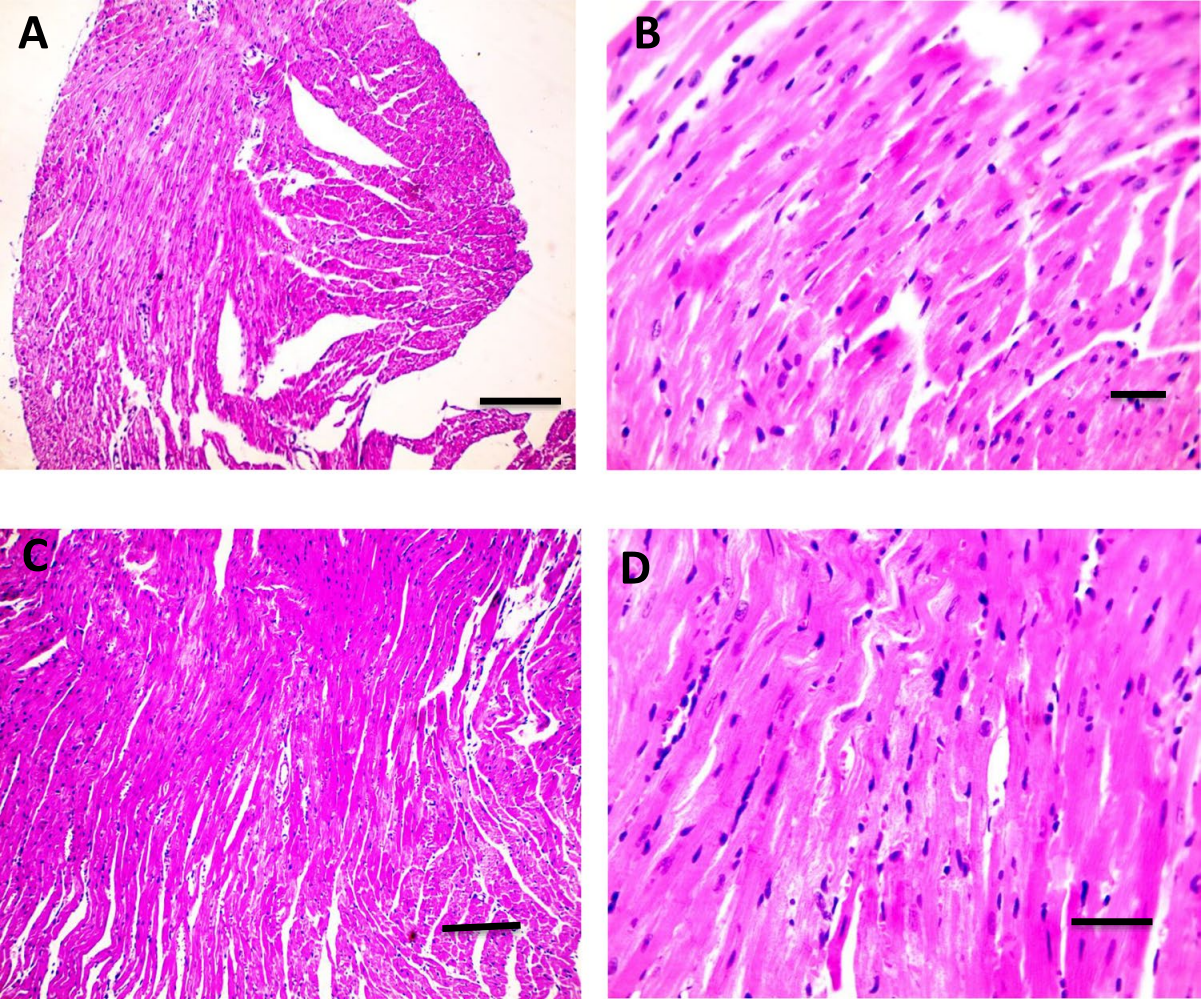

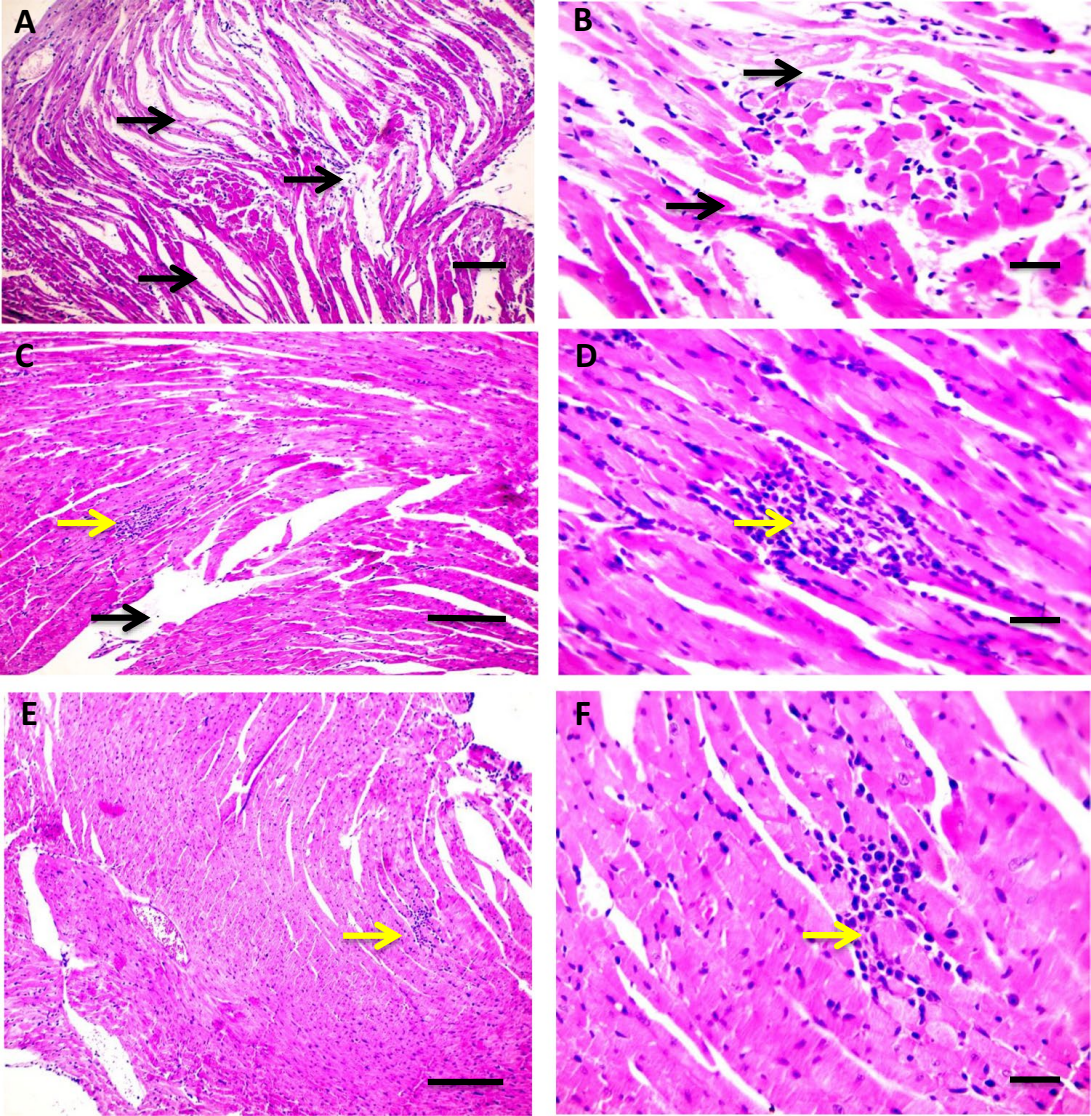

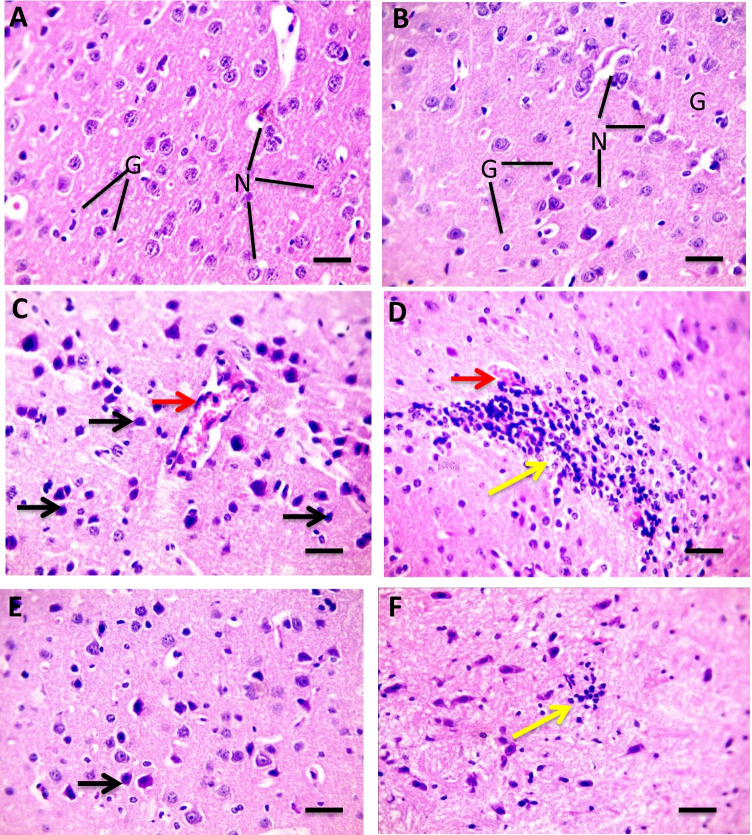

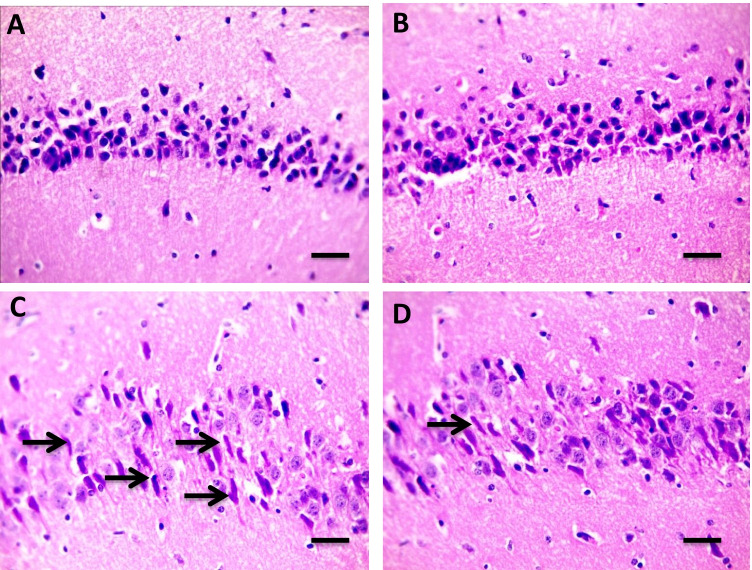

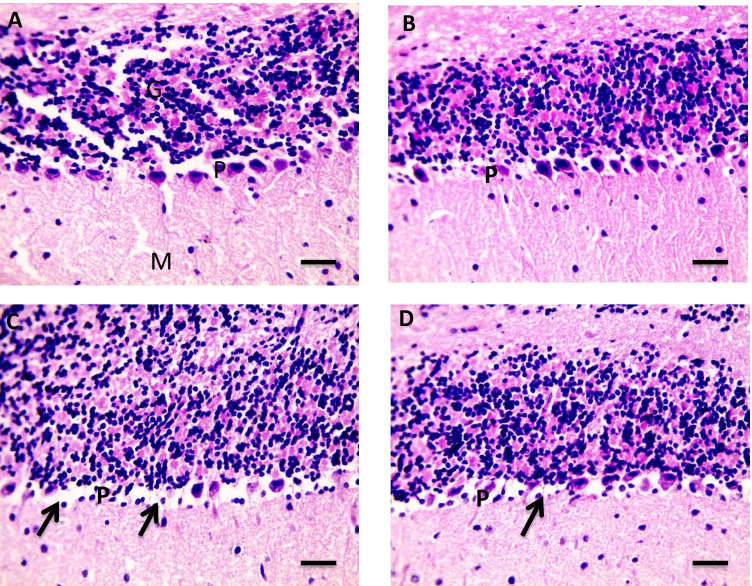
Effect of Flavo on heart and brain histopathological changes in Cyclo-injected mice. **4.1** Microscopic pictures of H&E stained cardiac sections showing a normal arrangement of cardiomyocytes in the control group (**A** and **B**) and the group received Flavo (**C** and **D**). (**A** and **C**) X:100 bar 100; (**B** and **D**) X:400 bar 50. **4.2** Microscopic pictures of H&E stained cardiac sections showing edema widely separate cardiomyocytes (black arrows) (**A** and **B**) with focal leukocytic cells infiltration (yellow arrow) in the intoxicated group (**C** and **D**). Meanwhile, few focal leukocytic cell infiltration (yellow arrow) are seen in the group received Flavo + Cyclo (**E** and **F**). (**A**, **C**, **E**) X:100 bar 100; (**B**, **D**, **F**) X:400 bar 50. **4.3** Microscopic pictures of H&E stained cerebral sections showing normal neurons (N) and glial cells (G) in the control group (**A**) and the group received Flavo (**B**). The congested blood vessel (red arrows), shrinkage, and degeneration in neurons (black arrows) besides marked glial cell proliferation (yellow arrow) (**C** and **D**) is seen in the intoxicated group. Milder neuronal changes and mild glial cell proliferation (yellow arrow) (**E** and **F**) are observed in the group that received Flavo + Cyclo. X:400 bar 50. **4.4** Microscopic pictures of H&E stained hippocampal sections showing normal pyramidal neurons in the CA3 region in the control group (**A**) and the group that received Flavo (**B**). Marked shrinkage and degeneration in neurons (black arrows) (**C**) are seen in the intoxicated group. Milder neuronal changes (black arrows) (**D**) are observed in the group that received Flavo + Cyclo. X:400 bar 50. **4.5** Microscopic pictures of H&E stained cerebellar sections showing normal granular (G), molecular (M), and purkinje (P) cell layers in the control group (**A**) and the group that received Flavo (**B**). Marked loss of purkinje neurons (black arrows) (**C**) is seen in the intoxicated group. Milder changes in purkinje neurons (black arrows) (**D**) are observed in the group that received Flavo + Cyclo. X:400 bar 50

As shown in Fig. 4 (4.1), H&E stained cardiac sections revealed a normal arrangement of cardiomyocytes in the normal control group (A&B) and the group received Flavo alone (C and D). Figure 4 (4.2) shows sections isolated from the Cyclo group with edema widely separated cardiomyocytes (black arrows) (A and B) with focal leukocytic cells infiltration (yellow arrow) in the intoxicated group (C & D). Meanwhile, focal few leukocytic cells infiltration (yellow arrow) was seen in a group protected with Flavo (E and F).

However, H&E stained cerebral sections showed normal neurons (N) and glial cells (G) in the control group (A) and the group received Flavo alone (B). Congested blood vessels (red arrows), shrinkage, and degeneration in neurons (black arrows), besides the marked glial cells proliferation (yellow arrow) (C & D), were seen in the diseased group. Milder neuronal changes and mild glial cells proliferation (yellow arrow) (E and F) were observed in the group received Flavo plus Cyclo (Fig. 4 (4.3)).

Hippocampal sections revealing normal pyramidal neurons in the CA3 region in the control group (A) and the Flavo (B) groups. Marked shrinkage and degeneration in neurons (black arrows) (C) were seen in the intoxicated group. Milder neuronal changes (black arrows) (D) were observed in the group received Flavo with Cyclo (Fig. 4 (4.4)).

The cerebellar section stained with H&E represented normal granular (G), molecular (M), and purkinje (P) cell layers in the control (A) and Flavo groups (B). Marked loss of purkinje neurons (black arrows) (C) was seen in the diseased group. Milder changes in purkinje neurons (black arrows) (D) were observed in the group received Flavo + Cyclo (Fig. 4 (4.5)).

### Influence of Flavo on immunohistochemical staining of cardiac and brain caspase-3

**Fig. 5:**
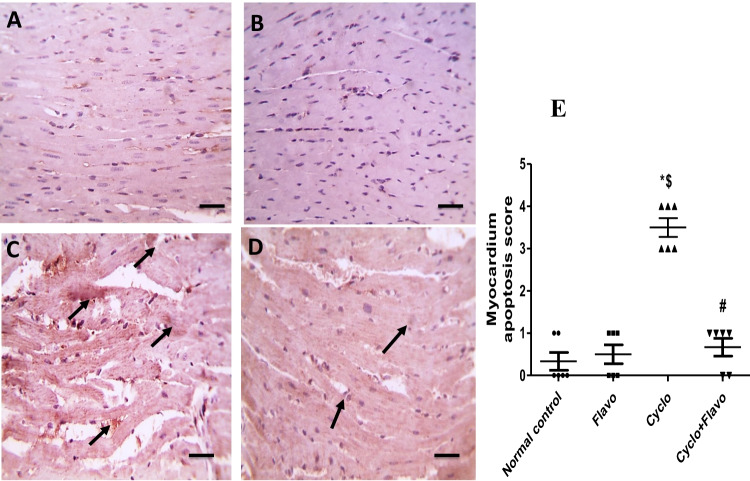

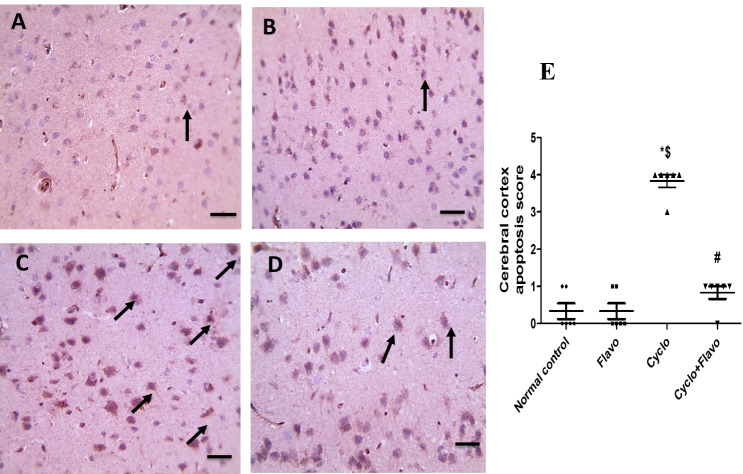
Effect of Flavo on heart and brain caspase-3 in Cyclo-injected mice. **5.1** Microscopic pictures of immunostained cardiac sections against caspase-3 showing negative staining in the control group (**A**) and group received Flavo alone (**B**). The marked positive brown expression appears in cardiac muscles in the Cyclo group (**C**) (black arrows). Caspase-3 positive expression markedly reduced in cardiac muscles in the group protected with Flavo compared to the Cyclo group (**D**). IHC counterstained with Mayer’s hematoxylin. X:400 bar 50. Scatter-dot plot representing the score of positive staining of cardiac caspase-3 (**E**). Data are expressed as mean ± SEM (*n* = 8). ^*, $, #^Significantly different from control, Flavo, and Cyclo groups, respectively. Statistical analysis was conducted using Kruskal–Wallis followed by Dunn’s multiple comparison test. **5.2** Microscopic pictures of immunostained cerebral cortical sections against caspase-3 showing negative staining in the control group (**A**) and Flavo group (**B**).The marked positive brown neuronal expression appears in the Cyclo group (**C**) (black arrows). The neuronal expression against caspase-3 reduced in the protected group with Flavo compared to the Cyclo group (**D**). IHC counterstained with Mayer’s hematoxylin. X:400 bar 50. Scatter-dot plot demonstrating the score of positive staining of the brain caspase-3 (**E**). Data are expressed as mean ± SEM (*n* = 8). ^*, $, #^Significantly different from control, Flavo, and the Cyclo groups, respectively. Statistical analysis was conducted using Kruskal–Wallis followed by Dunn’s multiple comparison test

In Fig. 5 (5.1), immunostained cardiac sections against caspase-3 showing negative staining in control (A) and Flavo alone groups (B). The marked positive brown expression appeared in cardiac muscles in the Cyclo group (C) (black arrows) compared with the normal heart group (E). Caspase-3 positive expression markedly decreased in cardiac muscles in the group protected with Flavo compared to the Cyclo group (D and E).

Cerebral cortical sections immunostained against caspase-3 in Fig. 5 (5.2) revealing negative staining in the control group (A) and Flavo group (B). Significant positive brown neuronal expression appears in the Cyclo group (C and E) (black arrows) when compared to the control group. The neuronal expression against caspase-3 decreased in the group protected with Flavo compared to the Cyclo group (D and E).

## Discussion

Cyclo is a major drug for cancer chemotherapy frequently used to treat breast cancer, but many side effects of Cyclo have been reported, such as cardio and neuroinflammation (Kurauchi et al. 2017; Flanigan et al. 2018). Thus, the natural antioxidant Flavo was selected to repair this inflammation due to its antioxidant and anti-inflammatory characteristics and scavenging free radicals with fewer side effects (El-sheakh et al. 2015). Flavo in a 1-g/kg/day dose revealed no-observed-adverse-effect level. In experimental studies, acute and subchronic toxicity indicated that Flavo has an optimal preclinical safety profile. This promising safety profile encourages the use of Flavo in humans. Clinical trials and a postmarketing study reported that Flavo has a significant efficacy in osteoarthritis and good overall and gastrointestinal tolerability, providing a potential therapeutic approach for acute and chronic inflammatory conditions (Abdelaziz et al. 2018). These characteristics were provided by its components, that is, bioflavonoids such as baicalin and catechin (Abdelaziz et al. 2018).

In this study, Flavo at a dose of 20 mg/kg for 14 days significantly improved the heart and brain weight indices; all estimated biochemical markers, such as LDH, CK-MB, NO, MDA, GSH, SOD, *TNF-α*, IL-1β, cTn-I, NF-κB, GM-CSF, and APP, and immunohistochemical examination of caspase-3 and histological examination of both heart and brain tissues.

Cyclo is considered one of the most common chemotherapeutic agents that induce vascular permeability and edema by changing vascular endothelial cell structure and permeability (Sagawa et al. 2020), and this was evaluated by this study by measuring both cardiac and brain weight indexes.

Compared to the normal group, there was a significant increase in the heart and brain weight index of the diseased group after the Cyclo injection (100 mg/kg, i.p.), whereas there was a significant decrease of these indices in the group treated with Flavo (20 mg/kg, orally) for 14 days. This in line with a previous study in which Flavo reduced prostate weight and inhibited prostate enlargement in a rat model with the same dose because Flavo can inhibit both the COX and *5-LOX* enzymes (Altavilla et al. 2012).

Oxidative stress is a phenomenon caused by an imbalance between the production and accumulation of oxygen reactive species (ROS) in cells and tissues and the ability of a biological system to detoxify these reactive products (Pizzino et al. 2017). These free radicals initiate chain reactions producing peroxidative breakdown of polyunsaturated fatty acids in the membrane bilayer. The interaction among oxygen-free radicals with polyunsaturated fatty acids has been known as lipid peroxidation and can be measured by malondialdehyde formation (PB et al. 1984). The accumulation of MDA depletes physiological antioxidants SOD and GSH. This study emphasizes the inflammatory response of Cyclo by raising MDA level compared with the control and Flavo groups. In Motawi et al., Cyclo was injected by dose of 200 mg/kg in rat models, which increased MDA in myocardium testicles and urinary bladder. Similarly, when it was injected at a dose 75 mg/kg i.p in rat, the brain exhibited a high level of MDA (Oboh and Ogunruku 2010). Interestingly, the protected group exhibited lower level of MDA and a higher level of SOD and GSH. The study of Abdelaziz et al. (2018) showed a marked decline in MDA level and relative increase in GSH and SOD when Flavo (20 mg/kg) for 16 days was used in an ovalbumin-induced mouse asthma model.

LDH is an important enzyme involved in energy production, and it is a good biomarker indicating cell damage (El-Agamy et al. 2017). Additionally, CK-MB is an enzyme found mostly in the heart and presents with significantly high amounts during cardiac damage (Cengiz et al. 2020).

In this study, there was a significant increase in LDH and CK-MB activities after only administration of Cyclo compared with the control group, which reflex the severe cardiotoxicity of Cyclo that is ensured by results of previous studies (El-Agamy et al. 2017; Gunes et al. 2017). The reason is that Cyclo is cardiotoxic by causing endothelial dysfunction and cardiomyocytes damage. Moreover, Cyclo causes lipid peroxidation, which disturbs endothelial cell permeability, resulting in elevated serum LDH and CK-MB levels (Cengiz et al. 2020). However, by the protective administration of Flavo with Cyclo, serum levels of LDH and CK-MB significantly diminished compared with the Cyclo-treated group.

Another biomarker contributing to Cyclo-induced cardiotoxicity is nitric oxide (NO). Although NO has an anti-inflammatory effect physiologically, NO plays an essential role in the pathogenesis of inflammation and oxidative stress in abnormal conditions (Papi et al. 2019). The current study found significantly high level of NO after a single dose of Cyclo compared with control, which aligns with previous studies (Iqubal et al. 2019b; Mansour et al. 2015). The explanation can be that acrolein, a metabolite of Cyclo, causes an increase in endothelial nitric oxide synthase (eNOS) monomers relative to eNOS dimers leading to eNOS uncoupling and, consequently, increase in nitric oxide and nitrative stress (Ismahil et al. 2011). By the protective administration of the Flavo with Cyclo, serum NO levels significantly decreased compared with the Cyclo-treated group. This result is in parallel with a previous study showing the ability of Flavo (20 mg/kg, i.p.) to improve NO levels in ovalbumin-induced asthma in mice (Abdelaziz et al. 2018).

Tumor necrosis factor-α (TNF-α) is an inflammatory cytokine that resulted from macrophages/monocytes during acute inflammation and is responsible for a diverse range of cell signaling events (Hartupee and Mann 2013). While IL-1β is a proinflammatory cytokine that has been involved in inflammation, pain, and autoimmune conditions (Mohan 2016). This work showed a significant increase in both heart and brain *TNF-α* and IL-1β by Cyclo (100 mg/kg, i.p.) compared with the control and Flavo groups, and this is in parallel with the study of Iqubal et al. (2020), which investigated that the Cyclo-induced neurotoxicity by the dose of 200 mg/kg in mice model through increasing the *TNF-α* and IL-1β significantly. Flavo decremented both levels in brain and heart homogenates in protected group compared with the Cyclo group, and this was in agreement with a study in which Flavo with dose (50, 100, 200 mg/kg; p.o.) in rat ischemic stroke model showed a significant decrease in *TNF-α* and IL-1β, and has a neuroprotective effect (Singh and Chopra 2014).

Cardiac Troponin I (cTn-I) represents a highly sensitive marker for myocardial cell death. This study shows the significant increase in cardiac troponin I with the Cyclo in heart homogenates compared to the control and Flavo groups. This is consistent with the previous study that showed high level of cTn-I following administration of Cyclo with a dose of 100 mg/kg in mice model (Iqubal, et al. 2019b). While Flavo with dose (20 mg/kg) alleviated cTn-I compared with the Cyclo group.

Nuclear factor kappa B (NF-κB) is considered a key transcription factor involved in many inflammatory disorders, such as cardiac disorder. It targets the proinflammatory genes and drives the expression of these genes leading to an elevation in production of cytokines and other proinflammatory proteins from cardiac tissue (Song et al. 2016). In the present study, there was a significant increase in heart NF-κB level after injection of Cyclo (100 mg/kg, i.p), and this coordinated with (Song et al. 2016) the work. Cyclo is considered a cardiotoxic agent that causes myocyte damage and extravasation of toxic metabolites (Dřímal et al. 2006). Otherwise, there was a marked decrease in heart NF-κB level after Flavo (20 mg/kg). This was illustrated by a previous study that referred to Flavo as an oral NF-κB inhibitor through its additional antioxidant effect (Vita et al. 2021). These previous results revealed for the first time the cardioprotective effect of Flavo by significantly improving previous cardiac toxicity biomarkers.

Granulocyte macrophage colony stimulating factor (GM-CSF) is now best viewed as a major regulator governing the functions of granulocyte and macrophage lineage populations at all stages of maturation. Hamilton and Hamilton (2002) study illustrated the crucial role of GM-CSF in inflammatory and autoimmune disease. The present work showed a significant increase in GM-CSF by Cyclo with dose (100 mg/kg) in brain homogenates compared to control and Flavo groups. Flavo with dose (20 mg/kg) alleviated brain GM-CSF compared to the Cyclo group.

APP services as a cell surface receptor in neurons at both presynaptic and postsynaptic sites, which are responsible for many physiological functions such as motility, cell growth, neurite outgrowth, neuronal adhesion, and cell survival through its large insoluble ectodomains; (sAPPα) and (sAPPβ) after breaking by α, β, and γ-secretase (Wolfe et al. 1999; Young-Pearse et al. 2007). APP was defined as a potentially effective biomarker indicating neuropathological alteration in the brain, and this was confirmed by the study of Araki et al. (2017). After injection of Cyclo (100 mg/kg, i.p), there was a significant increase in APP production level in comparison with the normal group, and this agreed with that of Butterfield et al. (2001) study who reported that the accumulation of free radical-oxidative stress and activation of neural lipid peroxidation is associated with the accumulation of APP, which worsens the neurodegenerative action of the Cyclo (Oboh and Ogunruku 2010; Flanigan et al. 2018). There was a significant decrease in APP level on administering Flavo (20 mg/kg, orally) for 2 weeks because Flavo can scavenge free radicals caused by Cyclo and blocks *5-LOX* pathway, in addition to decreasing phosphorylation/expression level of the amyloid protein and this integrated with work of (Bitto et al. 2017).

DNA damage and membrane peroxidation resulted from mitochondrial free radicals that promotes outer membrane permeabilization and facilitates the translocation of Bax and cytochrome C from the mitochondria to the cytosol with subsequent caspase activation. Caspase-3 promotes conformational changes and that initiates and executes apoptosis (Hochhauser et al. 2015). In the research of El-Agamy et al. (2017), the cardiac caspase-3 expression increased significantly after single Cyclo administration (200 mg/kg, i.p.) compared with the control. This is parallel with our work in which Cyclo elevated both cardiac and brain caspase-3 expressions, which was indicated by high immunoreactivity area. It was illustrated that Cyclo-induced cardiomyopathy via DNA intercalation, p53 protein activation, and ROS generation, which initiates the apoptotic pathway and then massive apoptosis of cardiomyocytes (El-Agamy et al. 2017).

Flavo administration decreased histological damage and caspase-3 in both heart and brain tissue in this study. This result is consistent with the work of Bitto et al. (2012) in which Flavo in a dose of 20 mg/kg improved pathological changes associated with sepsis in mice and apoptosis through inhibition of mitogen-activated protein kinases pathway, preserved β arrestin-2 expression, *TNF-α*, NF-κB, and increased IL-10 as well as lipoxin A4.

## Conclusion

**Fig. 6:**
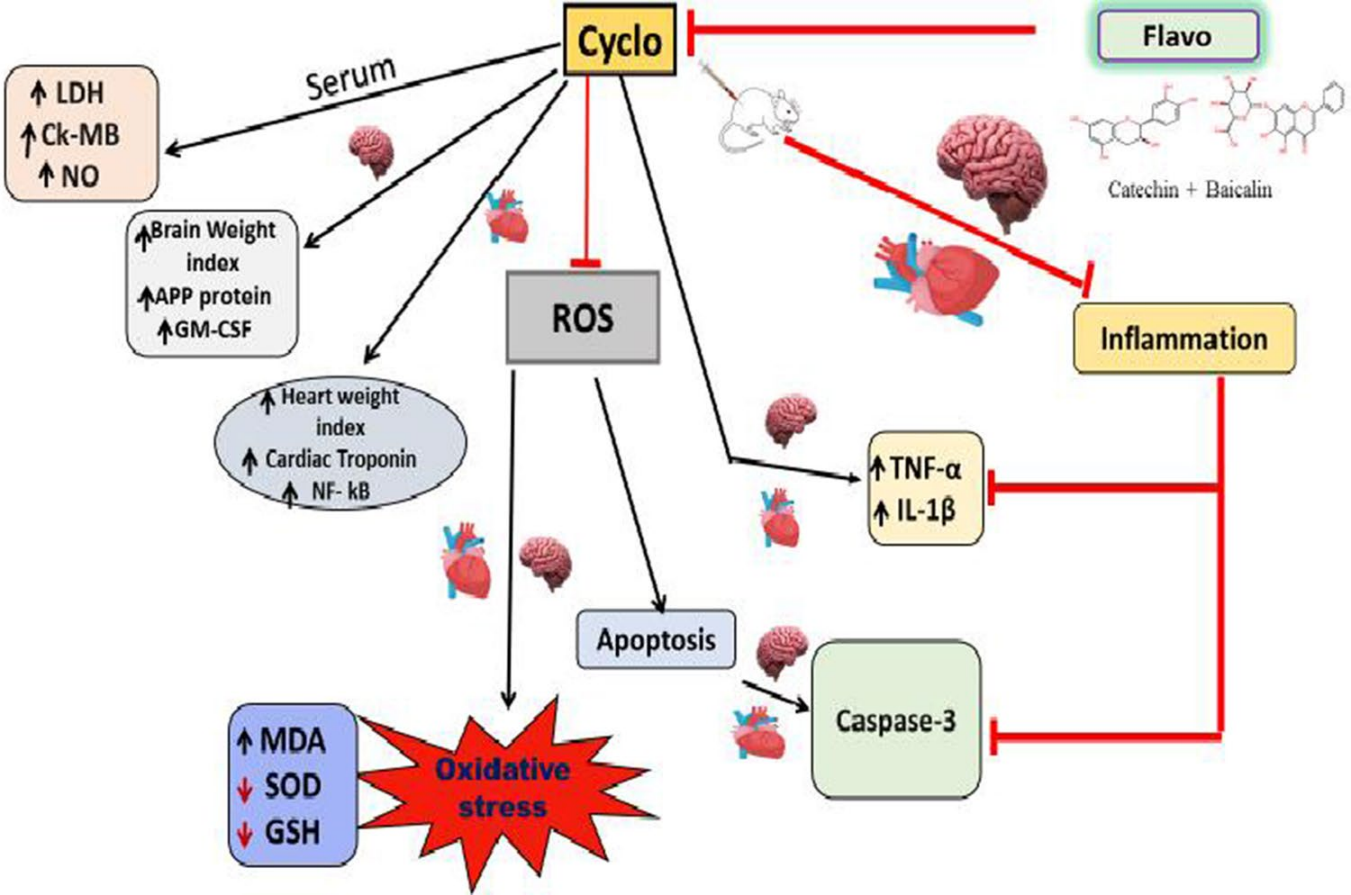
Graphical representation of the possible cascades or pathways involved in the protective effect of Flavo on Cyclo-induced cardio and neurotoxicities

Flavo administration attenuated cardiac/brain injury and ameliorated oxidative stress, inflammation, and apoptosis in the heart and brain, potentially by disrupting multiple signaling pathways mediated by GM-CSF/NF-κB, inflammatory cytokines (*TNF-α* and IL-1β), and apoptotic markers (caspase-3) (Fig. 6).

## Data Availability

The data that support the findings of this study are available on request from the corresponding author (Rania R. Abdelaziz). The data are not publicly available due to privacy.

## Abbreviations

*5-LOX*: Lipoxygenase
APP: Amyloid precursor protein
CK-MB: Creatine kinase
COX: Cyclooxygenase
Cyclo: Cyclophosphamide
cTn-I: Cardiac troponin I
Flavo: Flavocoxid
GM-CSF: Granulocyte macrophage colony-stimulating factor
GSH: Reduced glutathione
H&E: Hematoxylin and eosin
IHC: Immunohistochemistry
IL-1β: Interleukin-1β
i.p.: Intraperitoneal
LDH: Lactate dehydrogenase
MDA: Malondialdehyde
NF-κB: Nuclear factor kappa B
NO: Nitric oxide
PBS: Phosphate-buffered saline
ROS: Reactive oxygen species
SOD: Superoxide dismutase
*TNF-α*: Tumor necrosis factor-α
